# Photonuclear Alchemy: Obtaining Medical Isotopes of Gold from Mercury Irradiated on Electron Accelerators

**DOI:** 10.3390/molecules27175532

**Published:** 2022-08-28

**Authors:** Andrey G. Kazakov, Julia S. Babenya, Taisya Y. Ekatova, Sergey S. Belyshev, Vadim V. Khankin, Omar Albaghdadi, Alexander A. Kuznetsov, Illarion I. Dovhyi, Nikolay A. Bezhin, Ivan G. Tananaev

**Affiliations:** 1 Radiochemistry Laboratory, Vernadsky Institute of Geochemistry and Analytical Chemistry of the Russian Academy of Sciences (GEOKHI RAS), Kosygin St., 19, 119991 Moscow, Russia; 2 Department of Physics, Lomonosov Moscow State University (MSU), Leninskie Gory, 1, Bld. 2, 119991 Moscow, Russia; 3 Skobeltsyn Institute of Nuclear Physics, Lomonosov MSU, Leninskie Gory, 1, Bld.2, 119991 Moscow, Russia; 4 Department of Marine Biogeochemistry, Marine Hydrophysical Institute of the Russian Academy of Sciences, Kapitanskaya Str., 2, 299011 Sevastopol, Russia; 5 Department of Chemistry and Chemical Engineering, Sevastopol State University (SSU), Universitetskaya Str., 33, 299053 Sevastopol, Russia; 6 Department of Nuclear Technology, Far Eastern Federal University, Sukhanov Str., 8, 690091 Vladivostok, Russia

**Keywords:** ^198^Au, ^199^Au, photonuclear reactions, extraction chromatography, sorbents, benzo-15-crown-5, nuclear medicine

## Abstract

In our work, the photonuclear production of ^198,199^Au isotopes for nuclear medicine purposes was studied, and a method for their recovery from irradiated mercury was developed. The yields of the corresponding nuclear reactions were determined, and a comparison of various methods of obtaining gold radioisotopes was provided. New sorbents based on benzo-15-crown-5, which selectively binds gold, were studied, and the optimal conditions for Au recovery with a high degree of purification from mercury were found. It was established that, for the fast and quantitative recovery of Au isotopes, it was necessary to add at least 0.1 mg of the carrier. As a result, the developed method can be regularly used to obtain ^198,199^Au for the research of radiopharmaceuticals based on them.

## 1. Introduction

The radioactive isotopes ^198^Au (T_1/2_ = 2.7 d, 100% β^−^, E_γ_ = 411 keV) and ^199^Au (T_1/2_ = 3.1 d, 100% β^−^, E_γ_ = 208 keV) are considered promising for use in nuclear medicine in SPECT diagnostics [1,2,3,4,5,6]. ^198^Au can also be used for beta-therapy [7], in which case ^198^Au and ^199^Au become a theranostic pair. The advantage of using isotopes of gold lies in its chemical properties, thanks to which it becomes possible to synthesize the compounds unique for nuclear medicine. Moreover, one of the promising areas of modern research includes the use of ^198,199^Au-labeled nanoparticles for therapy 1.

**Figure 1:**
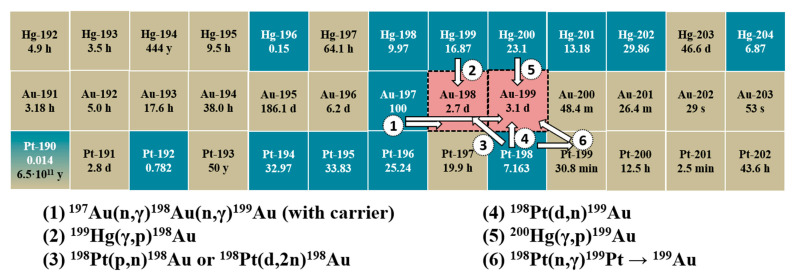
Studied ways of obtaining ^198,199^Au on a fragment of a nuclide chart. Aquamarine cells contain stable nuclei with their content in the natural mixture; sand cells contain unstable nuclei with their T_1/2_ (metastable nuclei are omitted).

^198,199^Au can be produced using reactors, cyclotrons, and electron accelerators; the studied methods are presented in Figure 1. Thus, both isotopes can be obtained by irradiating natural gold (100% ^197^Au) with neutrons; however, in these cases, a carrier is always present 1. To obtain carrier-free ^198^Au or ^199^Au, it is necessary to irradiate the nuclei of neighboring elements—Pt or Hg, which have six and seven stable isotopes in a natural mixture, respectively (see Figure 1). The production of ^198^Au and ^199^Au by irradiating ^198^Pt with protons, deuterons [8,9], or neutrons (in which case ^199^Pt, rapidly decaying into ^199^Au, was produced) were described [10]. A significant disadvantage of such production methods is the need to use extremely expensive targets made of enriched Pt to achieve Au isotopes with radioisotope purity sufficient for medicine. Finally, ^198^Au and ^199^Au can be obtained using the photonuclear method—by irradiating Hg with bremsstrahlung photons. This method also requires enriched targets (^199^Hg or ^200^Hg), but they are substantially cheaper than enriched Pt. In addition, for research, it is possible to irradiate ^nat^Hg or its compounds which have been actively disposed of in recent years and which are not difficult to find for free (for example, in old thermometers). There are very few data in the literature on the irradiation of mercury with bremsstrahlung photons. Thus, cross-sections per equivalent photon were calculated in [11], and the relative yields of nuclear reactions were determined in [12], but the values of the yields in units of Bq/μA·h were not presented. To summarize, the use of carrier-free ^198^Au or ^199^Au in nuclear medicine is significantly limited by the difficulties in obtaining them, and the further development of a photonuclear method for these purposes is one of the actual tasks.

In addition to obtaining Au isotopes, it is also necessary to develop a method for their recovery from irradiated targets. Sorption materials, widely used to solve the problems of radioanalytical chemistry [13,14], radiochemical technologies [15,16], and nuclear medicine [17], were studied in several works for the separation of Au and Hg. It was established that the *K_d_* of both elements in different media on cation- and anion-exchange sorbents was either very low or very high, which did not enable quantitatively separating them from each other [18,19,20,21,22,23]. Using commercial extraction chromatographic sorbents TEVA and LN (based on Aliquat 336 and HDEHP, respectively), it was possible to recover carrier-free Hg from macro-amounts of Au; however, 10–20% of Au remained on the column [24,25,26]. Sorbents Pb resin, DGA, and TK200 (based on 18-crown-6 derivative, diglycolamide, and trioctylphosphine oxide, accordingly) also made it possible to recover carrier-free Hg, but after this, it was impossible to desorb Au [27,28]. Moreover, there are currently no works devoted to the recovery of carrier-free Au from the macroquantities of Hg and, thus, the search for new methods remains an actual task.

The aim of our work was to determine the yields of photonuclear reactions leading to the production of ^198,199^Au as well as to develop a method for their quantitative recovery from the macro-quantities of irradiated Hg using new specially developed sorbents that selectively bind Au.

## 2. Results

### 2.1. Yields of Photonuclear Reactions on Hg Nuclei

To determine the yields of photonuclear reactions on Hg nuclei, 7.2 g of Hg were irradiated for 11 min. The obtained yields are presented in Table 1, and the yields of medical isotopes ^198^Au and ^199^Au were about 80 kBq/μA·h. Irradiation also produced other isotopes of gold—^200^Au with a yield 60 times greater and ^196^Au with a yield 10 times less. ^200^Au decays relatively quickly, however, ^196^Au decays twice as slowly as ^198,199^Au. Thus, as we mentioned above, in order to obtain ^198^Au and ^199^Au with high radionuclide purity, it is necessary to use enriched targets and optimal energy for irradiation.

### 2.2. Development of a Method of Recovery of Au Isotopes from Irradiated Hg Using Sorbents Based on Benzo-15-Crown-5

To develop a method of Au isotopes recovery, sorbents based on benzo-15-crown-5 (1,4,7,10,13-benzopentaoxacyclopentadecin, B15C5) and selectively binding gold, namely, B15C5-NB, B15C5-20, and B15C5-30, were used. Their preparation method and main properties are described in Materials and Methods, Section 5.3. To search for optimal conditions for recovery, we studied the sorption and desorption of Au in static and dynamic conditions and the sorption of Hg in dynamic conditions.

The distribution coefficients (*K_d_*) of Au on the studied sorbents in 0.1 to 3 M HCl solutions were determined earlier in our works [29,30]. However, in this work the separation was supposed to be carried out in more concentrated acids in order to avoid the hydrolysis of Au and Hg ions in solutions. Therefore, we studied the *K_d_* of Au in solutions from 5 to 9 M HCl, and the results are presented in Table 2 (see also Supplementary). It was established that, for B15C5-NB sorbent, the maximum *K_d_* was observed in 0.1 M HCl and was approximately 1400; for the B15C5-20 sorbent, in 3 M HCl, it was 1400; for B15C5-30 sorbent, in 9 M HCl, it was 2000. In these media, with the highest *K_d_* values, the sorption of Au in dynamic conditions was studied. It was found that, only when B15C5-30 is used, Au was quantitatively sorbed on the column, while in the case of B15C5-NB and B15C5-20, quantitative sorption from HCl solutions was impossible. 

When studying desorption from a column filled with B15C5-30, it was found that Au was not desorbed by HCl solutions of any concentration including ones with the lowest *K_d_*. At the same time, we determined that desorption was possible with HNO_3_ solutions with an acid concentration above 6 M, and with an increase in the acidity of the solution, an acceleration of Au desorption was observed. However, quantitative desorption under the studied conditions was not achieved; in each case, up to 20% of Au remained on the column. It was noted that, in the process of separation, part of the Au on the column (bright yellow) gradually turned pink or lilac, and during further elution, the colored section of the column did not move. This could be due to the formation of gold nanoparticles, for which such color is typical. To avoid their formation, aqua regia, which oxidizes nanoparticles, was subsequently used as an eluent, which finally made it possible to quantitatively desorb Au.

**Figure 2:**
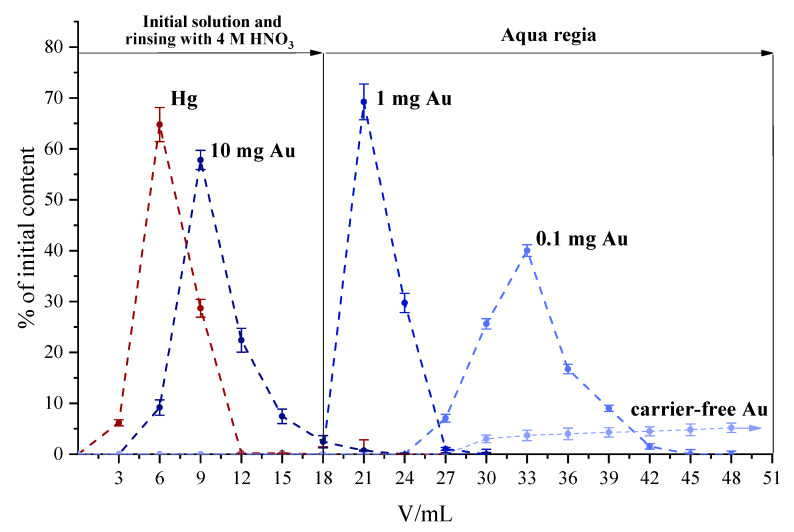
Elution curves of Hg and Au on a B15C5-30 column with the addition of various amounts of carrier.

As a result of the experiments described above, we found the optimal conditions for the separation of Au and Hg on B15C5-30 (Figure 2). Figure 2 demonstrate that the rate of desorption of Au from the column, using aqua regia as eluent, significantly depends on the amount of carrier added. Thus, with the preliminary addition of 10 mg of gold, Hg and Au did not separate. When 1 mg of carrier was added, 98% of Au was eluted from the column in 20 mL of aqua regia, and when 0.1 mg was added, in 30 mL. When Au without a carrier (about 10^−13^ g) was desorbed, only 35% of the sorbed Au was washed out of the column in 35 mL of eluate. Thus, for rapid and quantitative desorption, it is necessary to preliminarily add from 0.1 to 1 mg of the carrier.

**Figure 3:**
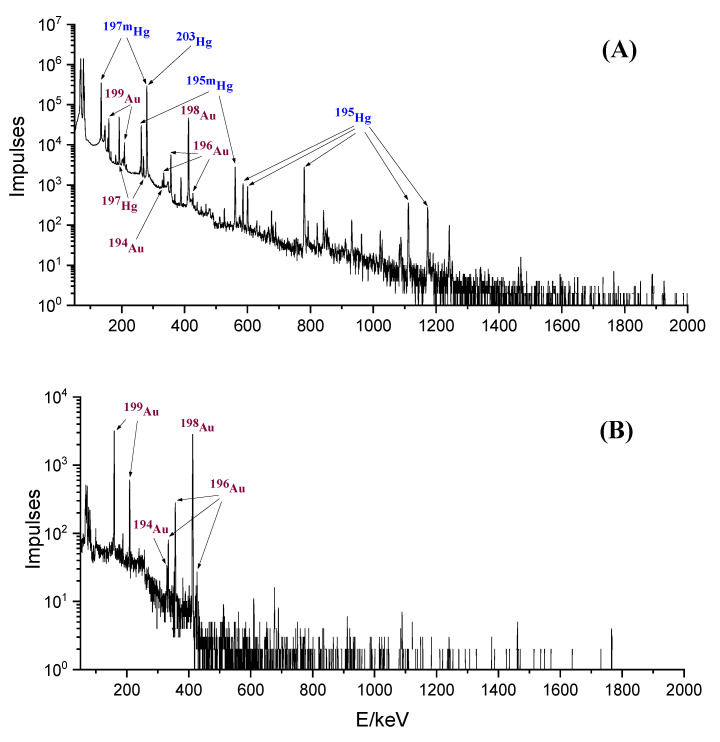
The spectrum of the irradiated target one day after the end of irradiation (**A**), and the spectrum of Au fractions separated from the column (**B**). The most intense peaks of the identified isotopes are marked.

During the prolonged registration of the gamma-ray spectra of the recovered Au fractions, the absence of peaks of Hg isotopes was established. The count rate of the most intense peak among them, 279 keV, was 107 cps before separation. The detection limit of this peak in measurement conditions was 7 × 10^−4^ cps and, thus, during separation, the Hg content decreased by at least 1.5 × 10^5^ times. To illustrate the degree of purification, the spectra of irradiated Hg_2_Cl_2_ and Au isotopes recovered from the target are presented in Figure 3, from which the absence of peaks of Hg isotopes is demonstrated.

## 3. Discussion

### 3.1. Comparison of Methods of Au Isotopes Production for Nuclear Medicine Purposes 

As we wrote in the *Introduction*, both carrier-free ^198,199^Au and ^198,199^Au with a carrier (to obtain or label gold nanoparticles) are used for the purposes of nuclear medicine. It should also be noted that short-lived gamma-emitter ^196^Au can be used for preclinical studies, as we used in this work. 

To evaluate the prospects of obtaining ^198,199^Au isotopes by irradiating Hg targets in electron accelerators, it is useful to compare various methods (using reactor, cyclotron, or electron accelerator) of obtaining Au isotopes. The choice of irradiation facility is influenced by the cross-section of the corresponding reactions, the availability of the facility, the target material, and the recovery method. To compare, we summarized the relevant data in Table 3, using the reaction cross-sections from the TENDL2021 library [31].

To obtain carrier-free ^198^Au, reactions 4, 5, and 7 from Table 3 can be used. The cross-sections for cyclotron reactions 4 and 5 reach 52 and 639 mbarn, which is higher than for reaction 7. Theoretical cross-section of reaction 7 is 0.3 mbarn according to TENDL, but theoretical values of photo-proton reactions in region of Hg nuclei are usually lower than real ones by order of magnitude [12], and, consequently, the real cross-section is approximately 3 mbarn. However, the need to irradiate very expensive ^198^Pt in the case of cyclotron reactions is their significant disadvantage. It can be concluded that reaction 5 is the most optimal for the production of ^198^Au for medical applications, while reaction 7 is more suitable for preclinical studies with small amounts of this isotope.

Reactions 6, 8, and 9 can be used to obtain carrier-free ^199^Au. The reaction cross-section of 9 is significantly higher than the others (3.96 barn), and this production method is obviously optimal for the production of large quantities of ^199^Au, although it requires enriched ^198^Pt. Reaction 8 can be considered suitable for the production of carrier-free ^199^Au for preclinical studies, since the cost of the target material is significantly lower. Finally, reaction 6 looks unpromising, since the target material is very expensive and the reaction cross-section is low for cyclotron ones.

The only way to obtain ^198,199^Au with a carrier is by irradiating ^nat^Au in a reactor (reactions 1, 2). The reaction 1 cross-section is high (107 barns), but the cross-section of reaction 2 is almost 250 times higher, which means that ^199^Au will also be formed during irradiation in couple with ^198^Au. On the other hand, both isotopes have similar nuclear properties, and it will not interfere with medical applications. Reaction 3, having a very high cross-section for electron accelerators (527 mbarn), can be used to obtain ^196^Au with a carrier in amounts sufficient for preclinical studies using relatively cheap targets.

Thus, the use of a reactor (reactions 1, 2, 9) and a cyclotron (reaction 5) is optimal for obtaining Au isotopes for use in nuclear medicine, while the use of an electron accelerator (reactions 3, 7, 8) is optimal for obtaining them for preclinical studies. Considering that research with Au isotopes is currently in the stage of radiopharmaceuticals development, the photonuclear method for obtaining Au isotopes for preclinical studies will contribute to their development and, consequently, to nuclear medicine.

### 3.2. Studied Sorbents and Au Isotopes Recovery

We studied the binding of Au to the new sorbents based on B15C5. It was found that the method of preparation of the sorbent and the content of B15C5 in it significantly affected the binding of Au. Thus, *K_d_* in statics for the studied sorbents could differ by almost two orders of magnitude. As for influence of diluent presence to sorption, it is well-known that crown-ethers can have different conformations, and not all of their conformers sorb metal cations well. The presence of a diluent allows the molecule of crown-ethers to change its conformation and often increase sorption. However, in the case of Au, the sorption mechanism was different, since it is likely that associates of B15C5 and H_3_O^+^ sorb [AuCl_4_]^−^. As a result, conformation of B15C5 did not affect sorption, and sorption of Au by B15C5-NB was lowest, since the concentration of B15C5 is lower than that of other studied sorbents.

Under dynamic conditions, B15C5-30, sorbent with highest content of B15C5, was the only one among those studied to quantitatively sorb Au. It was noted that there was an absence of Au sorption under dynamic conditions on B15C5-20, for which *K_d_* reached 300. This may be due to the slowly establishing equilibrium, which was not reached when the solution passed through the column.

## 4. Conclusions

We developed a method for separating Au isotopes from irradiated Hg compounds using the B15C5-30 sorbent. An unusual fact was that the amount of carrier significantly influenced the recovery of Au from Hg solution. We assume that this is due to the formation of an amalgam of gold during the dissolution of the targets, but, undoubtedly, the influence of the carrier needs further detailed study.

For the rapid and quantitative desorption of Au, at least 0.1 mg of the carrier must be added. Such an amount of Au is not dangerous for the human body when administered. In addition, we demonstrated a high degree of Au purification from Hg. For these reasons, it is possible to prepare and study radiopharmaceuticals based on ^198,199^Au obtained by our method. It is also worth noting that the process of dissolving the target, cooling the solution, and further recovery of Au takes no longer than 4 h. When using an electron accelerator located near the medical facility, our method will allow ^198,199^Au to be obtained without significant losses due to their decay.

As was written in the *Introduction*, when studying the behavior of Au on various sorbents, it was found that Au either does not bind to them or it binds so strongly that it cannot be quantitatively desorbed. We found a sorbent that not only sorbs Au quantitatively, but also from which it is possible to quickly and quantitatively desorb it. As a result, B15C5-30 can be further used not only to obtain medical isotopes of Au, but also be used in various analytical techniques.

## 5. Materials and Methods

### 5.1. Irradiation of Targets and Their Dissolution

All irradiations were performed using an RTM-55 electron accelerator of Lomonosov MSU with an electron beam energy of 55 MeV. The electron convertors were tungsten plates that completely overlapped the beam. Copper foils were placed before and after the target for calculating the passed charge using monitor reaction ^65^Cu(γ,n)^64^Cu. The following targets were irradiated in this work: Hg, Hg_2_Cl_2_, and Au.

To study the yields of photonuclear reactions, 7.2 g of Hg placed in a cylindrical polypropylene container with a diameter of 16.0 mm and a thickness of 2.8 mm were irradiated for 11 min. Hg did not occupy the entire internal space of the container, leaving a small air bubble. The thickness of the converter was 0.2 mm, and the average current was 16 nA. 

For radiochemical experiments including Au with a carrier, Au plates weighing 200 mg were irradiated for 1 h. During this irradiation, ^196^Au was produced with a high yield from the monoisotopic ^197^Au (see Table 3, reaction 3). To produce carrier-free Au isotopes, 1.5 g of Hg_2_Cl_2_ in a plastic container were irradiated for 7 h. In these cases, monitors were not used, and the current was not determined.

Au plates and Hg_2_Cl_2_ powder were dissolved in aqua regia, and aliquots of the resulting solutions were used in further experiments.

### 5.2. Determination of Yields of Nuclear Reactions and Detection of Elements during Separation by Gamma-Ray Spectrometry

The radionuclide composition of irradiated Hg was determined using a gamma-ray spectrometer with high-purity germanium detector GC3019 (Canberra Ind, Meridan, CT, USA). The calibration of count efficiency depending on the energy of the registered isotope was conducted using measurements of activity of certified point sources (^152^Eu, ^137^Cs, ^60^Co, ^241^Am) in the different location geometries of source and detector and was also modeled in GEANT4 software. The identification of a peak maximum in spectra was carried out using specially created for this purpose automatic system of spectrum record and analysis. Thus, spectra with a duration of 3.5 s each were saved into the data base, and analysis system allowed to summarize them and display total spectrum with assigned duration [32]. The registration of the spectrum began immediately after irradiation, with the target located 10 cm from the detector for the first 4 h, and then the spectrum was registered for another 16 d, while the target was at a distance of 5 cm from the detector. These experimental conditions made it possible to determine both short-lived and long-lived isotopes.

In radiochemical experiments, the content of Au and Hg was determined using a gamma-ray spectrometer Canberra GC1020 (Canberra Ind). To determine Au, peaks of ^196^Au (356 keV, 87%) or ^198^Au (412 keV, 96%) were used. Hg was determined using peak of ^197m^Hg (133 keV, 33%) and, after its decay, a peak of ^203^Hg (279 keV, 81%).

### 5.3. Studied Sorbents and Their Production

Sorbents were obtained as described in our works [29,30]. The prepared support was stirred in a rotary evaporator at a temperature of 60 °C for 2 h with a solution of B15C5 in a solvent in the presence of a nitrobenzene (NB) as diluent or without a diluent. Then, the solvent was distilled off by raising the temperature to its boiling point in the rotary evaporator. The obtained product was then dried at room temperature until constant weight.

Three sorbents were obtained: with NB as diluent, named B15C5-NB, and without diluent having 20% and 30% of B15C5, B15C5-20, and B15C5-30, respectively. The properties of the obtained sorbents are given in Table 4.

### 5.4. Study of Sorption and Desorption of Au under Static and Dynamic Conditions and Its Separation from a Solution Containing Macroquantities of Hg

The *K_d_* of Au on the studied sorbents were determined under stationary conditions using ^196^Au with a carrier. An aliquot of the solution of irradiated and dissolved Au plate was evaporated to dryness, redissolved in HCl, and mixed with the sorbent. The solution volume in each experiment was 2 mL, the mass of the sorbent was 150 mg, the Au content in each experiment did not exceed 1.5 mg, and the time of phases contact was 1 h. *K_d_* (mL/g) were calculated using the standard formula (equation. 1):

(1) $$K_{d}=\frac{A_{0}-A_{s}}{A_{s}}\cdot \frac{V}{m}$$

where *A*_0_ is the radioactivity before sorption (Bq), *As* is the radioactivity of the solution after sorption (Bq), *V* is the volume of the solution (mL), *m* is the mass of the sorbent (g).

The study of the sorption and desorption of Au and Hg under dynamic conditions and their further separation were carried out on columns with a volume of 2 mL and a diameter of 0.6 mm. The sorbents were preliminarily kept in dilute HCl for at least 24 h.

In each separation experiment, approximately 50 mg of irradiated Hg_2_Cl_2_ were dissolved in aqua regia, after which the resulting solution was diluted with water three times, leading to approximately 4.5 mL of solution. In the experiments with a carrier addition, an aliquot of the carrier solution was added before dilution. Then, 4.5 mL of the resulting solution was passed through a column with a sorbent, while Au was adsorbed on the column and Hg remained in the eluate. The column was then washed with 4 M HNO_3_ until Hg was completely removed, after which the Au was desorbed with aqua regia. During separation, fractions of 3 mL were collected. To determine the degree of purification of Au from Hg, the gamma-ray spectrum of the solution containing Au isotopes recovered from the column was registered for 24 h.

## Figures and Tables

**Table 1:** Radionuclide composition of irradiated Hg and experimental yields of reactions.

Isotope	T_1/2_	Main Reaction of Formation	Y_exp_, kBq/μA·h	Y_exp_, (kBq/μA·h)/ /(g/cm^2^)
^203^Hg	46.61 d	^204^Hg(γ,n)	200 ± 10	52.8 ± 2.6
^197^Hg	64.14 h	^198^Hg(γ,n)	7330 ± 432	1940 ± 114
^195^Hg	9.9 h	^196^Hg(γ,n)	1410 ± 40	372 ± 11
^200^Au	48.4 min	^201^Hg(γ,p)	5030 ± 376	1330 ± 99
^199^Au	3.14 d	^200^Hg(γ,p)	81.8 ± 4.1	21.6 ± 1.1
^198^Au	2.70 d	^199^Hg(γ,p)	77.3 ± 3.8	20.4 ± 1.0
^196^Au	6.17 d	^198^Hg(γ,pn)	7.05 ± 0.34	1.96 ± 0.09

**Table 2:** *K_d_* of gold(III) (mL/g) on the studied sorbents in HCl.

Sorbent	K_d_ in 0.1 to 3 M HCl [29,30]	K_d_ in 5 to 9 M HCl
B15C5-NB	320–1400	45–60
B15C5-20	200–1400	200–300
B15C5-30	1000–1400	1400–2000

**Table 3:** Cross-sections of nuclear reactions leading to the formation of Au isotopes according to TENDL2021 database [31].

	№	Reaction	Maximum Cross-Section, Mbarn	Incident Particle’s Energy at the Cross-Section Maximum, MeV
For production with carrier	1	^197^Au(n,γ)^198^Au	1.07 × 10^5^	thermal
	2	^198^Au(n,γ)^199^Au	2.51 × 10^7^	thermal
	3	^197^Au(γ,n)^196^Au	527	13.5
For carrier-free production	4	^198^Pt(p,n)^198^Au	52	10
	5	^198^Pt(d,2n)^198^Au	639	13
	6	^198^Pt(d,n)^199^Au	19	12
	7	^199^Hg(γ,p)^198^Au	0.3	28
	8	^200^Hg(γ,p)^199^Au	0.3	30
	9	^198^Pt(n,γ)^199^Pt→^199^Au	3.96 × 10^3^	thermal

**Table 4:** Properties of the obtained sorbents.

Sorbent	Diluent	The Content of B15C5 in the Sorbent, wt%	*Γ_t__h__eor_*, mg/g
B15C5-NB	NB *	7.41	56.8
B15C5-20	none	20.0	147
B15C5-30	none	30.0	221

* Concentration of B15C5 in diluent was 1 M.

## Data Availability

Not applicable.

## Supplementary Materials

The following supporting information can be downloaded at: https://www.mdpi.com/article/10.3390/molecules27175532/s1, Figure S1. Plot of *K_d_* determined earlier by atomic-emission spectroscopy (AES, from 0.1 to 3 M HCl), and in this work by radiometry (from 5 to 9 M HCl).
