# Supplementary material for: Photonuclear Alchemy: Obtaining Medical Isotopes of Gold from Mercury Irradiated on Electron Accelerators
Source: Molecules. 2022 Aug 28;27(17):5532. doi: 10.3390/molecules27175532 (PMC9457897; doi:10.3390/molecules27175532)
Supplement: Supplementary file 1 [file molecules-27-05532-s001.zip › molecules-1885318-supplementary.pdf]

Supplementary Materials:

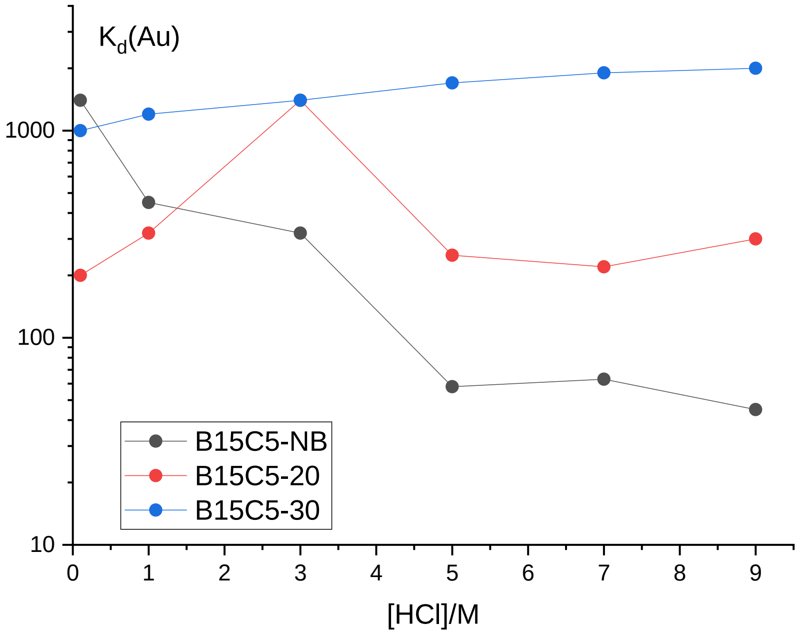

Figure S1. Plot of K_d determined earlier by atomic-emission spectroscopy (AES, from 0.1 to 3 M HCl), and in this work by radiometry (from 5 to 9 M HCl). The dependence of K_d on [HCl] may be related not only to the behavior of Au, but also to the fact that different amounts of Au were used for AES and radiometry detection, that, as determined during the separation of Au and Hg, may have an effect.
